# 苯达莫司汀联合利妥昔单抗一线治疗惰性B细胞非霍奇金淋巴瘤和套细胞淋巴瘤的真实世界研究

**DOI:** 10.3760/cma.j.issn.0253-2727.2023.04.012

**Published:** 2023-04

**Authors:** 凯欣 杜, 浩睿 申, 莉 王, 金花 梁, 佳竹 吴, 悦 李, 奕 夏, 华 尹, 建勇 李, 卫 徐

**Affiliations:** 南京医科大学第一附属医院（江苏省人民医院）血液科，南京 210029 The First Affiliated Hospital of Nanjing Medical University (Department of Hematology, Jiangsu Province Hospital), Nanjing 210029, China

惰性非霍奇金淋巴瘤（iNHL）具有恶性程度低、肿瘤生长缓慢、自然病程长、无法治愈且复发率高的特点[1]。套细胞淋巴瘤（MCL）兼具侵袭性淋巴瘤及惰性淋巴瘤的特征，预后较差[1]。长期以来，化学免疫治疗是iNHL患者的一线标准治疗[2]，对于年龄小于65岁的年轻MCL患者，接受利妥昔单抗联合含大剂量阿糖胞苷的方案后行自体造血干细胞移植可改善患者的生存[3]。但iNHL和MCL的中位发病年龄分别为67岁和60岁[1]，大剂量化疗带来的并发症对患者的生存质量有极大影响，如何选择疗效好、不良反应小的治疗方案是临床亟待探索的问题。

苯达莫司汀是一种兼具烷基化和抗代谢活性双重功能的抗肿瘤药物。STiL研究[4]和BRIGHT研究[5]–[6]显示，BR方案（苯达莫司汀+利妥昔单抗）治疗iNHL和MCL较RCHOP或RCVP方案有更佳的疗效和更好的安全性。我国苯达莫司汀获批适应证为既往接受过利妥昔单抗治疗的复发/难治的iNHL[7]，但国内尚无关于BR方案一线治疗iNHL和MCL的真实世界报道。本文探讨了本中心常规临床实践中应用BR方案一线治疗iNHL和MCL的疗效、安全性及预后因素。

## 病例与方法

1. 病例：收集2020年3月至2021年9月于南京医科大学第一附属医院（江苏省人民医院）血液科接受BR方案一线治疗的59例B细胞iNHL和6例MCL患者的临床资料。纳入标准包括：①所有患者均在治疗前进行组织活检，诊断符合2016年WHO淋巴肿瘤分类的标准[1]；②所有患者既往均未接受过治疗。排除标准包括：①3b级滤泡性淋巴瘤（FL）；②在BR方案基础上联合其他化疗药物及靶向药物。不同类型淋巴瘤的治疗指征参考中国恶性淋巴瘤诊疗规范（2015年版）[8]。所有患者均采用2014版Lugano分期标准进行分期。不同类型淋巴瘤采用不同的预后评分标准，包括FL国际预后指数（FLIPI）、FLIPI-2、黏膜相关淋巴组织淋巴瘤国际预后指数（MALT-IPI）、华氏巨球蛋白血症（WM）国际预后评分系统（IPSSWM）及简化版MCL预后评分（sMIPI）。

2. 治疗方案：利妥昔单抗375 mg/m^2^，第0天；苯达莫司汀90 mg/m^2^，第1～2天（年龄>75岁或ECOG评分≥2分的患者，苯达莫司汀剂量调整为70～85 mg/m^2^），28 d为1个周期。

3. 疗效评估：分别于治疗前、3个周期治疗后（中期评估）以及6个周期治疗结束后对患者进行疗效评估。如果患者存在骨髓受累，则需在疗效评估时复查骨髓涂片及活检。如果患者存在消化道受累，则需要完善胃镜及肠镜检查进行疗效评估。根据2014版Lugano疗效评定标准[9]，分为完全缓解（CR）、部分缓解（PR）、疾病稳定（SD）和疾病进展（PD）。对于WM，参考第六届瓦尔登斯特伦巨球蛋白血症国际研讨会更新的疗效标准[10]进行疗效评估。

4. 安全性评价：根据《常见不良反应事件评价标准（CTCAE）》5.0版[11]，对患者每个周期用药后发生的不良事件（AE）进行分级。

5. 随访：通过查阅门诊就诊记录、住院病历及电话进行随访，随访截止日期为2022年2月11日。无进展生存（PFS）时间定义为自患者开始治疗至疾病发生进展或死亡的时间；总生存（OS）时间定义为自患者开始治疗至因任何原因死亡或随访截止的时间。

6. 统计学处理：使用X-tile软件判断最佳截断值，采用SPSS 25.0和GraphPad Prism 8软件进行统计学分析。患者的临床特征、疗效和AE等资料采用描述性统计分析，偏态分布的定量资料以中位数（范围）表示，分类变量以例数（百分比）表示。生存分析采用Kaplan-Meier法。组间PFS和OS的比较采用对数秩检验。多因素分析采用Cox回归模型。*P*<0.05为差异有统计学意义。

## 结果

1. 临床特征：65例患者中FL 27例（41.5％）；边缘区淋巴瘤（MZL）24例（36.9％），其中黏膜相关淋巴组织边缘区淋巴瘤（MALToma）15例（62.5％）、淋巴结边缘区淋巴瘤（NMZL）9例（37.5％）；MCL 6例（9.2％），WM/淋巴浆细胞淋巴瘤（LPL）5例（7.7％），B淋巴细胞增殖性疾病，不能分类（BLPD-U）3例（4.6％）。中位年龄为55（24～80）岁，FL的中位发病年龄较MCL低（52岁对66岁）。男性占66.2％。以Ⅲ/Ⅳ期患者居多（57例，87.7％），但大多数患者体能状态良好，63例（96.9％）患者美国东部肿瘤协作组体能状况（ECOG PS）评分0～1分。根据疾病特有的预后评分系统对患者进行疾病分层，FL患者中FLIPI评分以中、高危居多，其中中危15例（55.6％），高危8例（29.6％）；MALToma患者中MALT-IPI评分中危11例（73.3％）。29例（44.6％）患者合并至少一种疾病，最常见的疾病是高血压（58.6％）和糖尿病（27.6％）。

2. 治疗方案和疗效评价：苯达莫司汀中位应用剂量为84.0（66.8～99.5）mg/m^2^，中位治疗6（1～6）个周期。6例患者停药或更换方案，其中3例患者因发生3～4级AE停药（1例为4级皮疹，1例为4级中性粒细胞减少，1例为肺部感染），2例患者因疾病控制不佳更换方案，1例患者因自身原因停止治疗。63例患者可评估疗效。49例（77.8％）患者获得CR，11例（17.5％）患者获得PR，总有效率（ORR）为95.2％（表1）。中位随访时间14.0（4.0～23.0）个月，中位PFS、OS时间均未达到（图1）。其中FL、MZL和MCL的ORR分别为96.3％、95.7％和80.0％。

**表1:** 苯达莫司汀联合利妥昔单抗方案一线治疗iNHL和MCL的疗效［例（％）］

疗效	总计（63例）	FL（27例）	MZL（23例）	MCL（5例）	其他（8例）
ORR	60（95.2）	26（96.3）	22（95.7）	4（80.0）	8（100）
最佳疗效					
CR	49（77.8）	21（77.8）	17（73.9）	3（60.0）	8（100）
PR	11（17.5）	5（18.5）	5（21.7）	1（20.0）	0（0）
SD	1（1.6）	0（0）	1（4.3）	0（0）	0（0）
PD	2（3.2）	1（3.7）	0（0）	1（20.0）	0（0）

注 iNHL：惰性非霍奇金淋巴瘤；MCL：套细胞淋巴瘤；FL：滤泡淋巴瘤；MZL：边缘区淋巴瘤；ORR：总有效率；CR：完全缓解；PR：部分缓解；SD：疾病稳定；PD：疾病进展

**图1:**
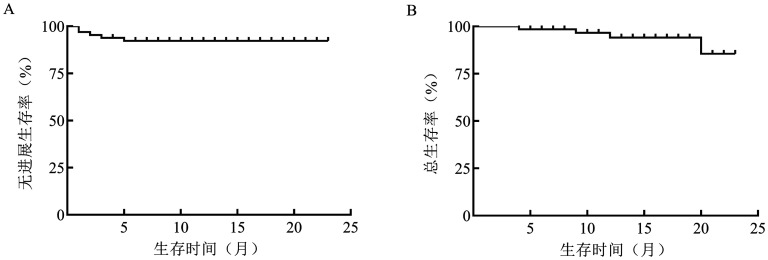
苯达莫司汀联合利妥昔单抗方案一线治疗惰性非霍奇金淋巴瘤和套细胞淋巴瘤的无进展生存（A）和总生存（B）曲线

3. 安全性评估：血液学不良反应的总体发生率（所有级别）为86.2％，最常见的血液学不良反应为CD4^+^T淋巴细胞减少（53例，81.5％），3～4级52例（80.0％）。中性粒细胞计数降低34例（52.3％），3～4级12例（18.5％）。发热性中性粒细胞减少6例（9.2％），均为3～4级。贫血11例（16.9％），3～4级0例。血小板减少20例（30.8％），3～4级7例（10.8％）。最常见的非血液学不良反应为轻度纤维蛋白原降低（34例，52.3％），3～4级1例（1.5％）。ALT/AST升高11例（16.9％），3～4级1例（1.5％）。肺部感染4例（6.2％），3～4级3例（4.6％）。皮疹6例（9.2％），3～4级5例（7.7％）。

53例患者在治疗期间定期进行淋巴细胞亚群检查。经过1个周期、4个周期及6个周期治疗后，分别有28例（52.8％）、39例（73.6％）及41例（77.4％）患者出现3～4级CD4^+^T淋巴细胞减少。维持治疗及治疗结束后，29例患者进行了淋巴细胞亚群的监测，其中23例（79.3％）患者的CD4^+^T淋巴细胞恢复。

4. 预后指标：本研究分析了年龄、性别、分期、B症状、ECOG PS评分、^18^氟-脱氧葡萄糖（^18^F-FDG）PET/CT最大标准摄取值（SUVmax）、β_2_-微球蛋白（β_2_-MG）、乳酸脱氢酶、治疗结束时是否达到CR等指标的预后价值。应用X-tile软件计算SUVmax的最佳截断值为13.0。单因素分析显示，年龄≤60岁（*P*＝0.029）、SUVmax≤13.0（*P*＝0.011）和治疗结束时达到CR（*P*<0.001）的患者具有较长的PFS时间，治疗结束时达到CR（*P*<0.001）与OS获益相关。将单因素分析中*P*<0.05的因素纳入多因素Cox回归分析中，仅SUVmax>13.0是PFS的独立预后因素（*HR*＝18.376，95％*CI* 1.171～288.322，*P*＝0.038）。对不同病理类型的患者进行亚组分析，FL患者中，SUVmax>13.0与较短的PFS时间（*P*＝0.002）和OS时间（*P*＝0.019）相关。MZL患者中，β_2_-MG水平高的患者具有较短的OS时间（*P*＝0.039）。

## 讨论

本研究回顾性分析了单中心65例一线应用BR方案治疗的iNHL和MCL患者的真实世界数据，结果表明，BR方案一线治疗iNHL和MCL疗效显著，安全性良好，ORR为95.2％，中位随访14.0个月，中位PFS、OS时间均未达到。RCHOP方案既往是大多数iNHL患者的首选，但在老年患者中，RCHOP方案的不良反应相对较大。BRIGHT研究比较了BR方案与RCHOP或RCVP方案一线治疗iNHL或MCL患者的疗效和安全性，结果显示BR组5年PFS率（*P*＝0.0025）及OS率（*P*＝0.5461）均明显优于RCHOP/RCVP组，并且显示出更持久的疾病控制[5]–[6]。安全性方面，BR方案组药物过敏（如皮疹）的发生率高，而RCHOP/RCVP方案组周围神经不良反应及脱发更常见，BR方案不良反应可控，耐受性可。STIL研究对比了BR和RCHOP方案一线治疗初治iNHL和MCL患者的疗效和安全性，结果显示，BR方案组的PFS（*P*<0.001）明显优于RCHOP组，同时，BR方案的耐受性优于RCHOP方案[4]。国内一项真实世界回顾性研究分析了BR方案治疗iNHL的临床疗效，该研究共纳入73例初治和复发/难治iNHL患者，结果显示，接受BR方案的患者ORR为79.5％，其中CR率为37.0％，PR率为42.5％，中位PFS时间为12.1个月，中位OS时间为15.5个月[12]。尽管CSCO指南已经将BR方案列为FL等iNHL的一线推荐方案，但大多数BR方案的一线数据基于欧美人群[13]，国内的临床经验相对欠缺。

苯达莫司汀2019年在国内上市，临床实践中应用BR方案一线治疗iNHL和MCL的时间较短。本研究的中位随访时间为14.0个月，期间出现2例PD，其中1例为FL患者，在第2个周期BR方案治疗过程中发生了组织学转化，重新病理活检诊断为高级别B细胞淋巴瘤；另1例为MCL，在2个周期后PD；另有1例MALToma患者在3个周期后评估为SD；其余患者在随访期间均达PR或CR，中位PFS及OS时间均未达到，提示疗效较好。但本研究为回顾性，并未对同期应用RCHOP或RCVP方案治疗的患者进行统计学分析，存在一定的偏倚。

BR方案安全性较好，本研究中最常见的3～4级AE是CD4^+^T淋巴细胞减少，但感染率较低，仅3例患者出现3～4级肺部感染，其中仅1例患者因肺部感染停药。本研究对53例患者进行了规律的淋巴细胞亚群检查，发现在BR方案治疗期间，随着苯达莫司汀药物累积，CD4^+^T淋巴细胞进行性减少，而完成诱导治疗后进入随访阶段的大部分（79.3％）患者的CD4^+^T淋巴细胞计数恢复。

此外，本研究还进一步分析了可能有意义的预后指标，单因素分析显示年龄≤60岁、SUVmax≤13.0和治疗结束时达到CR与PFS获益相关，多因素Cox回归分析表明SUVmax>13.0是PFS的独立预后因素。亚组分析显示，SUVmax>13.0的FL患者的生存明显劣于SUVmax≤13.0的患者，中位PFS、OS时间分别为3个月和12个月，但由于本研究中SUVmax>13.0的FL患者仅有2例，因此可能存在较大的偏倚。Strati等[14]的研究表明，对于接受非蒽环类方案治疗的FL患者，SUVmax>18.0提示预后较差。2020年Mondello等[15]发现初诊时SUVmax≥13.0的FL患者应用RCHOP方案较BR方案可获得更高的CR率。因此，SUVmax>13的患者肿瘤侵袭性更强，可能更加推荐应用RCHOP方案治疗，但需进一步行前瞻性临床研究进行验证。

综上所述，一线BR方案治疗iNHL和MCL患者具有良好的疗效和安全性，本研究为我国iNHL和MCL患者一线应用BR方案提供了一定的依据。但由于是回顾性分析，样本量小，且未与同期应用RCHOP或RCVP方案治疗的患者进行对比分析，存在一定的局限性。此外，由于苯达莫司汀在国内上市时间较短，仍需进一步延长随访时间以获得更准确的预后信息。
